# Effects of self-controlled feedback on learning range of motion measurement techniques and self-efficacy among physical therapy students: a preliminary study

**DOI:** 10.1186/s12909-025-06908-2

**Published:** 2025-02-28

**Authors:** Ryohei Yamamoto, Takaki Imai, Yushin Yoshizato, Kazunori Akizuki

**Affiliations:** 1 Department of Rehabilitation, Kumamoto Health Science University, 325, Izumi-machi, Kita-ku, Kumamoto-shi, Kumamoto, 861-5598 Japan; 2 Department of Rehabilitation, Kyushu University of Nursing and Social Welfare, 888, Tomino, Tamana, Kumamoto, 865-0062 Japan; 3 Department of Physical Therapy, Mejiro University, 320, Ukiya, Iwatsuki-ku, Saitama-shi, Saitama, 339-8501 Japan

**Keywords:** Range of motion measurement, Physical therapy students, Self-controlled feedback, Self-efficacy

## Abstract

**Background:**

Measuring range of motion (ROM) accurately using a universal goniometer or visual estimation is challenging for physical therapy students. Self-controlled (SC) feedback, where learners decide whether to receive feedback, can enhance learning and foster self-efficacy (SE) by promoting self-regulation. However, the impact of SC feedback on skill acquisition in ROM measurement technique and SE in physical therapy students remains unclear. This study investigates the effects of SC feedback on skill acquisition in ROM measurement techniques and students’ SE.

**Methods:**

Thirty physical therapy students were quasi-randomly assigned to an SC group, which chose feedback during practice, or a Yoked (Yk) group, which received feedback based on the SC group’s schedule. A goniometric measurement task, in which participants measure the ROM of left knee flexion using a universal goniometer, and a visual estimation task, in which they estimate it visually, were set as the learning tasks. After a pretest, they completed the practice (3 trials × 4 blocks) followed by short-term retention test (STRT) and LTRT (LTRT). All tests consisted of 3 trials. Measurement accuracy and time were used as test performance for both tasks. SE of ROM measurements was measured before the start of each test using a 10-point Likert scale. Feedback related to measurement errors were provided during practice in line with each group’s conditions.

**Results:**

The SC group maintained high feedback frequency (80.0 ± 30.3%) during the practice. Both groups improved measurement accuracy and reduced time for goniometric measurement and visual estimation tasks, but no significant group differences were found. Goniometric accuracy exceeded visual estimation in both groups. SE before the pretest did not correlate with pretest accuracy. However, SE before the STRT correlated with accuracy at that time in both groups. In the SC group, SE before the LTRT test was related to the accuracy at the STRT.

**Conclusion:**

SC feedback did not demonstrate superior effectiveness, but external feedback improved ROM measurement accuracy and reduced measurement time. Moreover, SE after the practice was temporarily associated with accuracy, suggesting a potential link between SE and performance in skill acquisition.

## Background

When the joint range of motion (ROM) is restricted owing to aging, neurological diseases, or orthopedic diseases, all aspects of daily life are affected. Previous studies have demonstrated that when the joint range of motion is restricted in the knee joint, the propulsive force during walking is reduced, whereas the risk of falling increases [1–5]. Furthermore, in activity daily living, which requires a large range of joint motion, such as squatting and putting on underwear, if the joint range of motion is restricted, the performance of the movement will be hindered [6]. In addition, ROM limitation is associated with a decline in technical motor skills and sports performance [7–9] and has been reported to increase the risk of muscle injury [10–12]. Therefore, ROM measurement is an important testing technique for medical professionals to appropriately understand a patient’s condition and determine the effectiveness of treatment.

The measurement methods include measurements based on radiographs [13–15], electronic goniometers [16, 17], universal goniometers [18–19], and visual estimation [20–22] and equipment such as three-dimensional motion analysis devices [23]. Although radiograph-based measurements are considered the gold standard, they are difficult to obtain routinely in clinical settings because of the high cost of equipment, limited measurement locations, and radiation exposure [24]. Therefore, in clinical practice, visual estimation, which allows measurements to be performed without tools, or measurements using inexpensive universal goniometers are commonly used [25].

ROM measurements obtained using a universal goniometer have been found reliable when performed by the same examiner on the same day and within the same session [26–31]. Akizuki et al. compared the accuracy of ROM measurements between physical therapy students and physical therapists with clinical experience and reported that ROM measurement accuracy increased with experience [32]. Although the accuracy of visual estimation also improves with experience, it is less reliable than measurements obtained using a universal goniometer [14, 33]. On the other hand, Blonna et al. reported that visual estimation by experienced therapists has higher measurement reliability and validity than universal goniometric measurements [25]. Following these findings, for physical therapy students with poor measurement skills, visual estimation is expected to be less accurate than goniometric measurement. Furthermore, as the accuracy of angle measurements improves with practice, the accuracy of visual estimation is also expected to improve, thereby narrowing the gap between the two methods. However, the difference in accuracy between visual estimation and goniometric measurement by physical therapy students as well as how each change with practice remain unclear. If these measurements, which are commonly used in clinical settings, are inaccurate, the assessment of severity and prediction of prognosis will also be inaccurate, which may hinder the selection of appropriate treatment methods. Improving and mastering these customary measurement techniques from student years is important for providing appropriate medical care to patients in clinical settings after obtaining qualifications as a physical therapist.

One factor that facilitates the acquisition of various skills is feedback provided during practice. Feedback refers to information provided externally as instructions regarding exercise results, such as knowledge of results and knowledge of performance [34]. Akizuki et al. (2020) reported that practicing using a feedback device improved the accuracy of ROM measurements using a universal goniometer in physical therapy students [35]. The study showed that providing feedback on all 15 practice trials improved measurement accuracy in physical therapy students compared with a control group that received no feedback. Various feedback methods exist, and in recent years, the effectiveness of self-controlled (SC) feedback has attracted attention. SC feedback allows learners to choose or control their feedback schedules. Previous studies investigating the effects of SC feedback have typically included a yoked group. For example, each participant in the SC group receives feedback on the trials of their choice, whereas participants in the yoked group receive feedback on the same trials requested by the participants in the SC group. The purpose of this procedure is to control the frequency and timing of feedback. The effectiveness of SC feedback in learning various motor skills has been reported [36–41].

Although goniometric measurement and visual estimation both involve the motor element of manipulating the subject’s limbs, the task is highly sensory in that the correct angle must be visually judged. SC feedback is also effective in mobilization for the lumbar spine, a physical therapy technique [42]. When practicing a mobilization task, learners learn the strength of force applied to their fingers, i.e., their sense of touch, so the task is considered to have a strong sensory element, and SC feedback is thought to be effective for learning such tasks as well. Several studies investigating self-controlled learning adopt Zimmerman’s self-regulation of learning model [43] as the theoretical framework to understand the potential benefits of self-controlled feedback [36, 44, 45]. Zimmerman categorizes self-regulation into three phases: forethought, performance control, and self-reflection. The forethought phase includes self-regulatory processes that enable behavior prior to the action and beliefs, such as SE, whereas the performance control phase occurs during the actual execution of the behavior. Finally, the self-reflection phase includes numerous self-regulatory processes that occur after the performance. Information from previous motor performance is used for subsequent adjustments, and the three phases function cyclically, changing the SE and performance in the process. SE is defined as the perception of one’s ability to successfully perform a required behavior in a particular situation [46]. In the field of medical education, Morton et al. (2006) state that final-year medical students often do not self-assess themselves properly and are unaware that their skills are below the standard [47]. Even for physical therapy students who have learned the ROM measurement method, SE does not reflect the accuracy of ROM measurement [48], indicating that students do not accurately recognize their performance. Practice under SC feedback conditions is expected to affect not only skill acquisition but also SE, and several studies have investigated the relationship between SE and performance after self-regulation feedback. Ste-Marie et al. (2013) investigated children learning trampoline skills and showed that children in the SC group performed better than children in the Yk group [49]. A hierarchical multiple regression analysis was used to show that self-control as well as self-efficacy at retention significantly predicted performance at retention [45]. In addition, Kok et al. (2020) reported that practice with SC feedback improved SE [44].

By utilizing the three phases of self-regulation in the learning model, practicing under SC feedback condition is predicted to promote physical therapy students’ acquisition of ROM measurement and visual estimation skills. In addition, during the process, students may correctly recognize their skills and obtain SE that reflects their measurement accuracy. However, to date, there have been no reports on the effects of practicing SC feedback on ROM measurements, a basic technique in physical therapy. Therefore, this study investigated the effects of SC feedback on the skill of ROM measurements using a universal goniometer and visual estimation in physical therapy students. In addition, we observed a relationship between SE and measurement accuracy before and after practice. We formulated the following three hypotheses:

1. Physical therapy students who have the opportunity to choose when to receive feedback will more effectively produce ROM measurement techniques learning compared to students who do not have the opportunity.
2. Visual estimation by physical therapy students is less accurate than measurements made using a universal goniometer; however, accuracy improves with practice.
3. The measurement accuracy and SE using the universal goniometer, which were unrelated before practice, become correlated after practice through practice using SC feedback.

## Methods

### Study design and participants

This study was approved by the Ethics Committee of the Kumamoto Health Science University (approval ID: 22007). Before the study, participants were asked for their cooperation after they received a verbal explanation of the contents of the study and how to handle the results, based on the research manual. Participants were requested to sign consent forms of their free will.

We conducted a prospective, 2 (feedback: SC versus Yk) × 2 (measurement method: goniometric measurement versus visual estimation) factorial, quasi-randomized study. The participants were 30 physical therapy students (male: 18, female: 12, age: 19.6 ± 0.83 years) at a four-year university in Kumamoto Prefecture, Japan, which included 22 s-year students and 8 third-year students. Undergraduate students were recruited voluntarily between May and November 2023. By the time of their participation, they had completed lectures and exercises on joint ROM measurements; however, they had never performed ROM measurements on patients during training at a hospital.

A power analysis was conducted to estimate the sample size using G*Power 3.1.9.7. We used repeated measured analysis of variance (ANOVA), within-between interaction with an α error level probability of 0.05, and a power (1-β error probability) of 80%. The medium effect size Cohen was set to f = 0.25. The analysis described above revealed that a total sample size of 40 was required for this study. However, we were only able to collect data from 30 participants due to the limited number of individuals who met the inclusion criteria during the recruitment period.

### Measuring equipment

The participants used a universal goniometer with a handle length of 30 cm and angle marks at 1° increments. In this study, the reference ROM values for the knee joint were measured using ARMS software (ATR-promotions, Kyoto, Japan) based on the measured values obtained by two electronic accelerometers (TSND151, AMWS020; ATR-promotions, Kyoto, Japan). The values measured by the accelerometer were transferred to a computer connected via Bluetooth, and the joint angles were calculated based on these values and displayed on a screen in real time. Previous studies have confirmed the reliability of knee joint angle measurements using electronic accelerometers for various movements such as walking, climbing stairs, and jumping. In particular, high reliability has been reported for measurements in the sagittal plane [50]. For left knee flexion measurements, an accelerometer was attached to the lower limb 5 cm from the lateral epicondyle of the femur along the line connecting the greater trochanter and lateral epicondyle of the femur, whereas the other accelerometer was attached 5 cm from the fibular head along the line connecting the fibular head and lateral malleolus. These landmarks were identified by palpation. In addition, electronic accelerometers were installed using the method described by Yamamoto et al. [48]. All accelerometers were secured with surgical tape, whereas elastic bandages were used to prevent slippage. The joint angles calculated from the accelerometer measurements and saved at a sampling frequency of 1,000 Hz were displayed on a monitor using ARMS software to notify individuals who underwent ROM measurements of the joint angles.

### Tasks

#### Goniometric measurement task

**Fig. 1:**
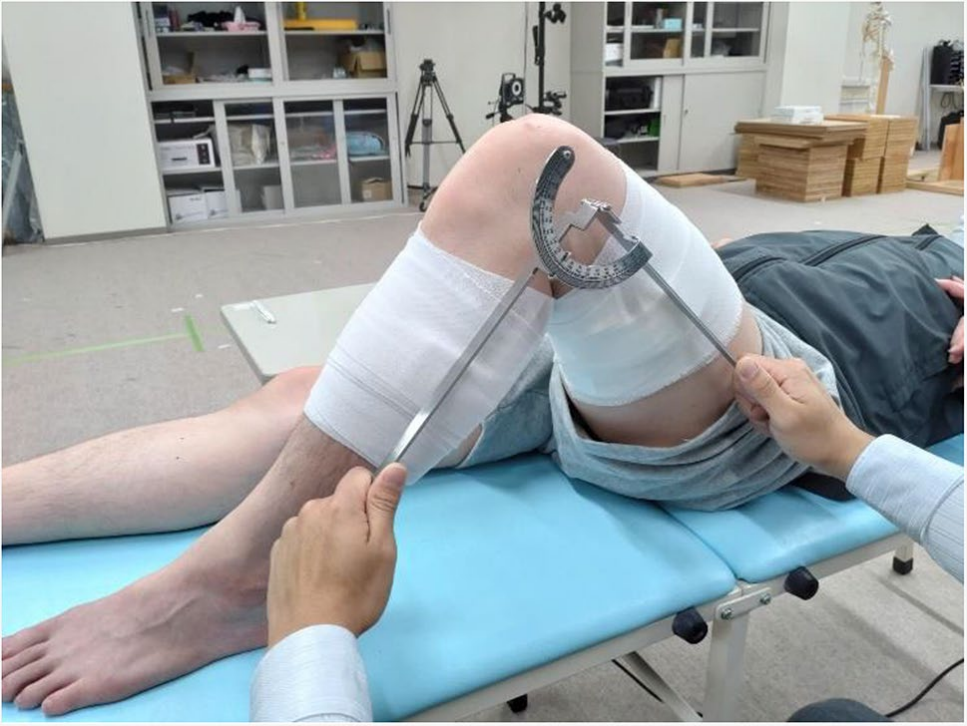
Setup during the ROM measurement

In this study, with reference to the study by Akizuki et al., the task was to measure the ROM of the left knee joint of the subject in the supine position [35]. The landmarks for the ROM measurement of the knee joint were the greater trochanter, lateral epicondyle of the femur, fibular head, and lateral malleolus. The angle formed by the line connecting the greater trochanter and lateral epicondyle of the femur and the line connecting the fibular head and lateral malleolus was measured in 1°steps by the participant using a universal goniometer (Fig. 1). No instructions were provided on how to locate the landmarks by palpation, passively bend the joint, or apply a goniometer to the measurement site. During the trials, the right knee joint flexion was set to angles of 60° ± 10°, 75° ± 10°, and 90° ± 10°. The person to be measured watched the display of the software, and when the set angle was reached, announced that “the knee will not bend any further” and resisted the force being applied to flex the knee by the participant. Once the movement stopped, participants measured the joint angle in 1° increments using a universal goniometer. Furthermore, as soon as they completed the measurements, they declared “done” and reported the measurements to the experimenter. The experimenter recorded the time at which the measurement was completed using the time-recording function of the ARMS software. The person to be measured was the same for all participants.

#### Visual Estimation task

The visual estimation task was performed in the same manner as the goniometric measurement task, with only the method of measuring the joint angles being changed. In this task, the subject passively flexed the subject’s left knee joint, and when it stopped, observed it directly and estimated the joint angle in 1° increments without using a device such as a universal goniometer.

### Procedure

**Fig. 2:**
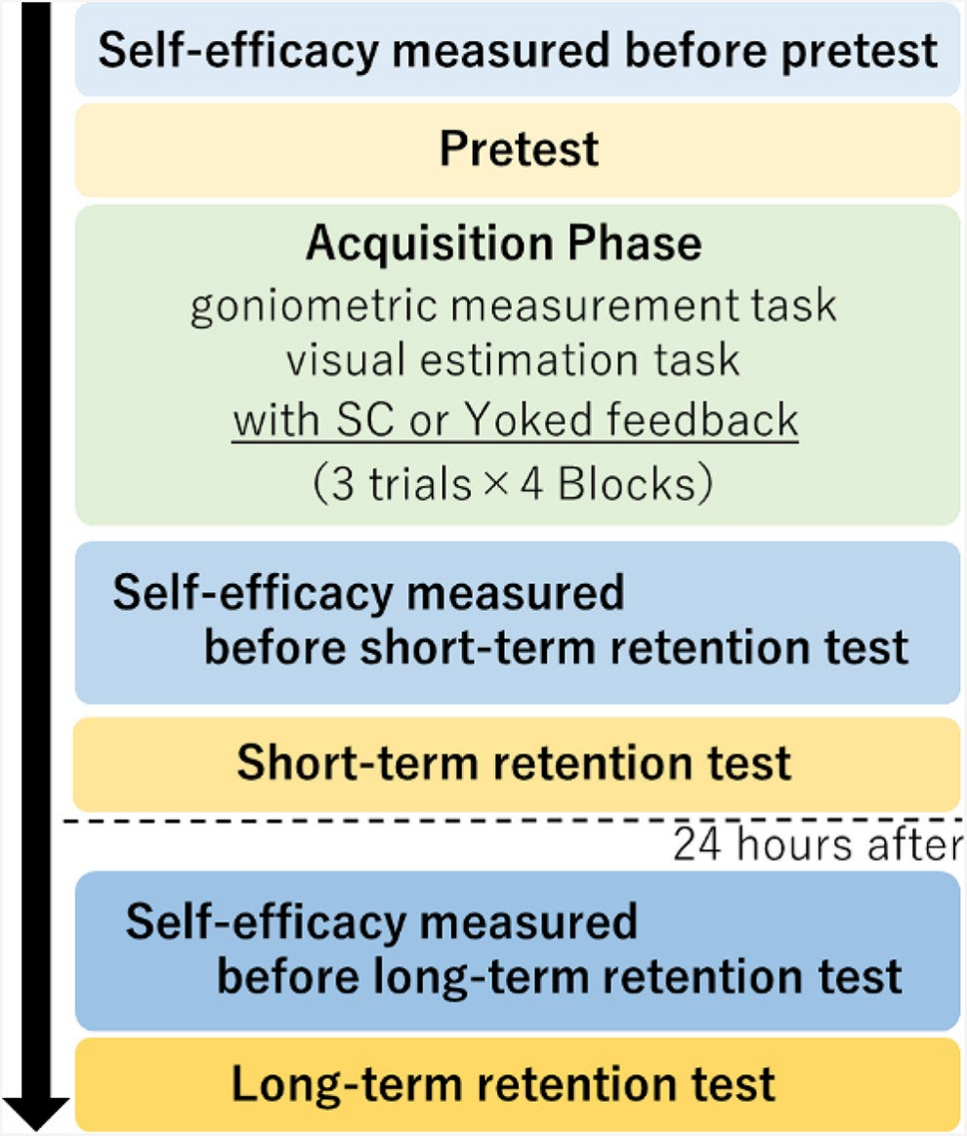
Measurement procedure

The participants were quasi-randomly assigned to two groups, a self-control group (SC group) and a Yoked group (Yk group), except that there were an equal number of second- and third-year students in each group. The number of students in each group was the same. All participants participated in the two-day experiment according to their assigned groups (Fig. 2). First, SE was assessed before the pretest and prior to the practice. Each item was rated on a 10-point Likert scale, where 1 indicated a lack of confidence and 10 indicated complete confidence. Many studies on SE have used Likert scales, though the number of points can vary, typically ranging from 5 to 10 [51, 52]. According to Gist et al., when baseline SE is high, there is limited room for improvement [53]. Therefore, a 10-point Likert scale was chosen, consistent with the method used to measure SE for shoulder joint ROM assessment [54]. After measuring SE, three trials [60° ± 5°, 75° ± 5°, and 90° ± 5°; in random order) of both the visual estimation and goniometric measurement tasks were conducted as a pretest. The practice was conducted after the pretest. In the practice, 12 trials (3 trials × 4 blocks) were conducted, and the three joint angles were randomly set for three trials in each block. During practice, after the visual estimation task, the participants performed the goniometric measurement task while keeping their left knee joint. There was a 30-second break between each trial and a one-minute break between each block. In this study, we designed the learning task and experimental procedure based on the method of Akizuki et al. [35] Their study adopted the same goniometry task as our study and included 15 practice trials. However, we included an SE measure, a visual estimation task, and a short-term retention test that were not included in their study, which increased the time required to measure one participant on the first day. The number of practice trials was slightly reduced to 12 to reduce the burden on participants.

During practice, participants in the SC group chose whether to receive feedback at the end of each trial and were given feedback on the goniometric measurement task only during the preferred trials. The feedback information was the joint angle displayed on the screen when the subject declared completion of the measurement and the experimenter verbally communicated it to the subject in 1° increments. In contrast, the participants in the Yk group were not given the opportunity to make a choice and received feedback only on trials in which the corresponding participants in the SC group received feedback. In addition, the participants in the Yk group performed the tasks in the same order as the corresponding participants in the SC group.

Ten minutes after practice ended, SE was measured again, followed by a short-term retention test (STRT) in the same manner as in the pretest. Approximately 24 h after the end of the STRT, SE was measured, and subsequently, the long-term retention test (LTRT) was conducted in the same manner as the pretest. The STRT indicates the immediate effects of practice, while LTRT reflect the degree of motor learning. Furthermore, the participants were not provided feedback on either test.

Figure 2 represents the schematic diagram of the measurement procedure. SE of the ROM was measured before each test. In all the tests, the goniometric measurement and visual estimation tasks were measured in the same manner, with three trials each. In the practice, the participants practiced each task for a total of 12 trials (3 trials × 4 blocks). In addition, feedback was provided according to the condition of the assigned group only during practice, and no feedback was given to all participants during all tests.

### Data processing and statistical analysis

First, since feedback frequency in the SC group varied based on participants’ choices, we calculated it as the ratio of the number of feedback instances to the total number of trials in a block. Next, we set measurement accuracy and measurement time as indicators of ROM measurement technique. The measurement accuracy indicates the accuracy of the technique, whereas the measurement time indicates the smoothness of the technique. The main results of this study were the measurement accuracy and measurement time of the goniometric measurement and visual estimation tasks in each test and the SE for ROM measurement before each test. First, the accuracy of the goniometric measurement task was calculated as the absolute error between the joint angle measured by an electronic accelerometer and that measured by the student using a universal goniometer. Similar to the goniometric measurement task, the measurement accuracy of the visual estimation task was defined as the absolute error between the joint angle measured using the electronic accelerometer and the student’s visual estimation. Next, for both the goniometric measurement and visual estimation tasks, the measurement time was defined as the time from the experimenter’s declaration of the start of measurement to the participant’s declaration of the completion of measurement and was measured in milliseconds. These results were averaged for each task in the pretest, STRT, and LTRT. SE was defined as the real number on a ten-point Likert scale as the SE of the ROM measurement before each test measurement.

Statistical analysis was performed using IBM SPSS ver.29 (IBM Corp., NY, USA). First, an analysis was conducted to determine the degree of learning for each task based on the differences in the accuracy of goniometric measurements and visual estimation and the difference in the way feedback was provided. A three-way analysis of variance was conducted with measurement accuracy as the dependent variable and testing, feedback, and measurement method (3 × 2 × 2) as factors. A sub-test using the Bonferroni method was performed if a significant main effect or interaction was observed. A similar analysis was conducted regarding the measurement time of the goniometric measurement method and visual estimation. Finally, to confirm whether the SE of each group was related to the measurement accuracy of the goniometric measurement task, the measurement accuracy of the goniometric measurement task of each test and the SE before each test were subjected to Spearman correlation analysis. A similar analysis was performed on the measurement accuracy of the visual estimation task. In all analyses, a risk rate of less than 5% was considered statistically significant.

## Results

### Feedback frequency

The feedback frequencies from block 1 to block 4 were 86.7 ± 21.1%, 75.6 ± 34.4%, 75.6 ± 36.7%, and 80.0 ± 30.3% (mean ± standard deviation) in order.

### Measurement accuracy

**Fig. 3:**
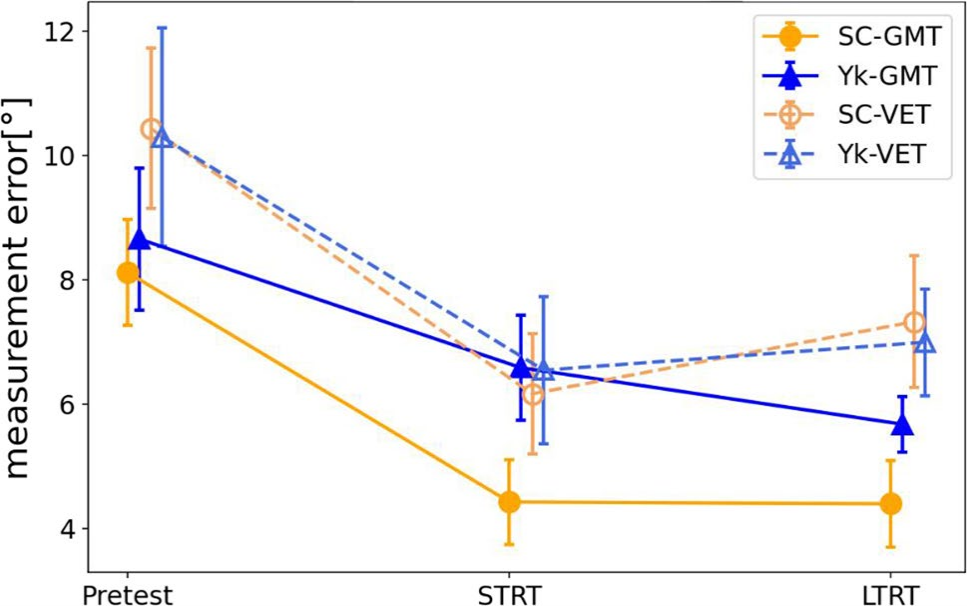
Measurement accuracy. This figure shows the change in measurement accuracy for the two tasks for each group. The error bars in the graphs represent the standard error. SC-GMT, goniometric measurement task of self-controlled group, Yk-GMT: goniometric measurement task of the yoked group, SC-VET: visual estimation task of self-controlled group, Yk-VET: visual estimation task of the yoked group; STRT, short-term retention test; LTRT: Long-term retention test

The results of a three-way ANOVA with the measurement error of each test as the dependent variable and 3 (test) × 2 (feedback) ×2 (measurement method) as independent variables revealed that among the within-subject factors, there was a significant main effect of the test (F(2, 56) = 13.756, *p* < 0.001, η_p_^2^ = 0.329) (Fig. 3). As a subtest, the STRT and LTRT were significant compared to the pretest using the one-way ANOVA and the Bonferroni method, with the measurement accuracy of each test as the dependent variable and the test as the independent variable (both *p* < 0.001). Furthermore, among the within-subject factors, there was a significant main effect of the measurement method, and the measurement error in the goniometric measurement task was significantly lower than that in the visual estimation task (F(1, 28) = 8.343, *p* = 0.007, η_p_^2^ = 0.230). Although there was a main effect of feedback (F(1, 28) = 0.644, *p* = 0.429, η_p_^2^ = 0.022), no interaction effects of feedback and test (F(2, 56) = 0.698, *p* = 0.757, η_p_^2^ = 0.010), no interaction effects of feedback and measurement method (F(1, 28) = 1.396, *p* = 0.247, η_p_^2^ = 0.047), no interaction effects of test and measurement method (F(2, 56) = 0.776, *p* = 0.465, η_p_^2^ = 0.027 ), and no interaction between feedback, measurement method, and test (F(2, 56) = 0.136, *p* = 0.873, η_p_^2^ = 0.005) were determined.

### Measurement time

**Fig. 4:**
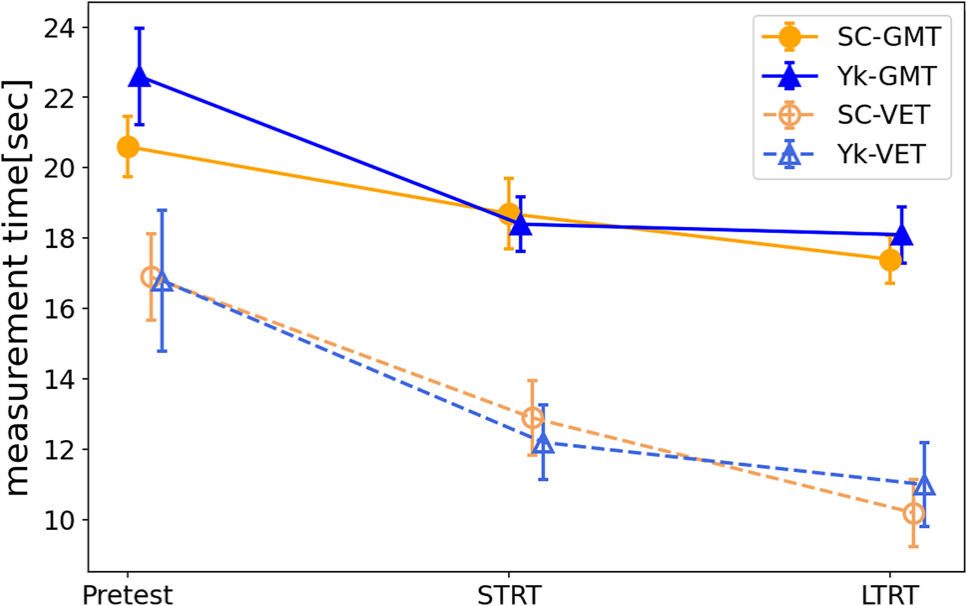
Measurement time. This figure shows the change in measurement time for the two tasks for each group. The error bars in the graphs represent the standard error. SC-GMT, goniometric measurement task of self-controlled group, Yk-GMT: goniometric measurement task of the yoked group, SC-VET: visual estimation task of self-controlled group, Yk-VET: visual estimation task of the yoked group, STRT: short-term retention test, LTRT: long-term retention test

The results of a three-way ANOVA with the measurement time of each test as the dependent variable and 3 (test) × 2 (feedback) × 2 (measurement method) as independent variables revealed that among the within-subject factors, there was a significant main effect of test (F(2, 56) = 31.683, *p* < 0.001, η_p_^2^ = 0.531) (Fig. 4). As a subtest, the short-term and long-term retention tests were significant compared to the pretest, using the one-way ANOVA and the Bonferroni method, with the measurement accuracy of each test as the dependent variable and the test as the independent variable (both *p* < 0.001). Furthermore, among the within-subject factors, there was a significant main effect of the measurement method, and the measurement time in the visual estimation task was significantly shorter than that in the goniometric measurement task visual estimation task (F(1, 28) = 110.547, *p* < 0.001, η_p_^2^ = 0.798). Although there was a main effect of feedback (F(1, 28) = 808.225, *p* = 0.731, η_p_^2^ = 0.004), no interaction effects of feedback and test (F(2, 56) = 0.698, *p* = 0.502, η_p_^2^ = 0.024), no interaction effects of feedback and measurement method (F(1, 28) = 0.542, *p* = 0.468, η_p_^2^ = 0.019), no interaction effects of test and measurement method (F(2, 56) = 2.597, *p* = 0.083, η_p_^2^ = 0.085), and no interaction between feedback, measurement method, and test (F(2, 56) = 0.528, *p* = 0.593, η_p_^2^ = 0.019) were determined.

### Correlation between SE and measurement accuracy

**Fig. 5:**
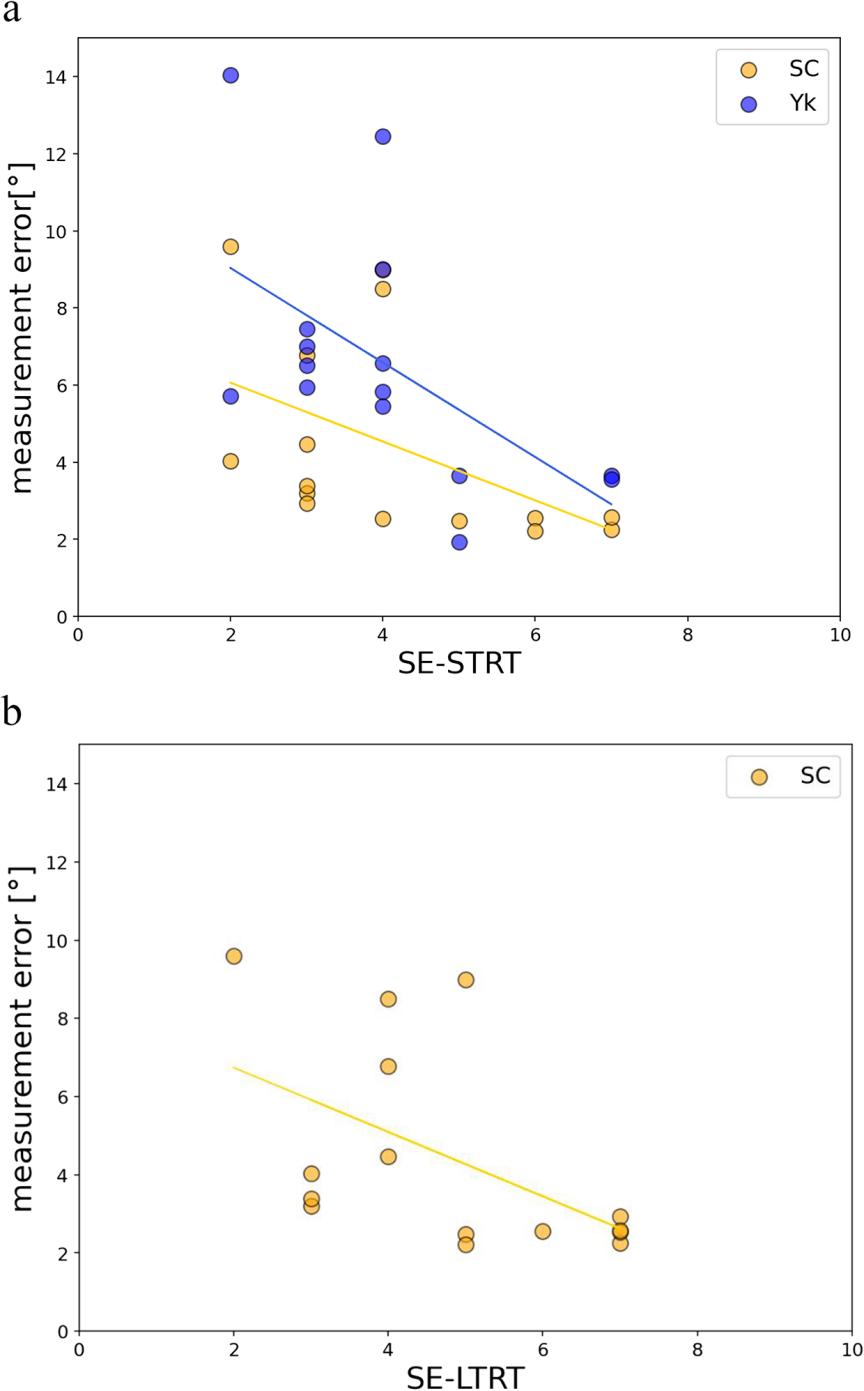
(**a**) Relationship between measurement accuracy of the short-term retention test and SE before the short-term retention test for each group. (**b**) Relationship between the short-term retention test measurement accuracy and SE before the long-term retention test in the SC group. In **a**, the vertical axis shows the absolute error between the actual ROM and the measured value of the short-term retention test, whereas the horizontal axis shows self-efficacy. For the dotted and regression lines, yellow represents the SC group, whereas blue represents the Yk group. In **b**, the vertical axis shows the same as in **a**. The horizontal axis shows the SE at the long-term retention test, which represents the SC group. SE-STRT: self-efficacy at the short-term retention test; SE-LTRT: self-efficacy at the long-term retention test.

A correlation analysis between SE before each test and the measurement accuracy of the goniometric task of each test in the SC group revealed that SE before the STRT had a moderately significant negative correlation with the measurement accuracy of the STRT (ρ= -0.675, *p* = 0.006) (Fig. 5a). In addition, SE before the long-term retention test had a moderately significant negative correlation with the measurement accuracy of the STRT (ρ= -0.636, *p* = 0.011) (Fig. 5b). Subsequently, we conducted a similar analysis in the Yk group and found that SE before the STRT had a moderately significant negative correlation with STRT measurement accuracy (ρ=-0.635, *p* = 0.011) (Fig. 5a). Additionally, a correlation analysis between the measurement accuracy of the visual estimation task for each test and the SE before each test in both groups showed no significant correlation.

## Discussion

This study investigated the effects of SC feedback on physical therapy students’ skills for ROM measurement and visual estimation employing a universal goniometer used by medical professionals such as doctors and physical therapists. In addition, we observed a relationship between SE and measurement accuracy before and after practice. As the results, the measurement accuracy of goniometric measurements and visual estimation improved and the time required for measurement was reduced in both the SC and Yk groups through practice. While the measurement accuracy of visual estimation was lower than that of angle measurement, the measurement time of angle measurement was longer than that of visual estimation. No differences were detected between the SC and Yk groups. Before practice, SE did not reflect the measurement accuracy of ROM measurements made by physical therapy students, but after practice, it began to reflect the measurement accuracy. In the SC group, SE on the day after practice showed a correlation with the measurement accuracy immediately after practice. To our knowledge, this is the first study to examine the effects of SC feedback on the ROM measurement practice.

First, regarding measurement accuracy, the results of this study showed that regardless of how feedback was given, practicing with feedback improved the accuracy of ROM measurement using a goniometer in physical therapy students. Moreover, this skill was maintained the day after practice. These results revealed that practice using feedback improved the accuracy of ROM measurements in physical therapy students. Akizuki et al. reported that a feedback device using an electronic goniometer improved students’ measurement accuracy when practicing passive ROM measurements of the knee joint using a goniometer [35]. The tasks used in this study could be characterized as perceptual tasks in which the angle was visually judged. Sietz et al. examined the effect of feedback on learning in the visual direction-discrimination task and reported that learning occurred only when feedback was used [55]. Feedback related to learners’ responses has been reported to be similarly effective in learning a vernier discrimination task [56], demonstrating its effectiveness in visual perceptual tasks. However, the effectiveness of SC feedback was not demonstrated in the present study. Although the number of practice trials in this study was 12, which was not large, one study that verified the motor learning effect of self-selecting the objects to be used reported that it was effective after 15 trials [57]. Furthermore, task-relevant choices, such as feedback, have been reported to be more effective than task-irrelevant ones, such as the items used [58]. Considering these factors, it should have been easier to observe the effects of SC feedback in this study, but this was not the case. St Germain et al. stated that feedback characteristics are more important determinants of motor learning than the presence or absence of selection [59]. In this study, as the SC group used feedback frequently, the Yk group received frequent feedback during practice as well. Janelle et al. reported that participants in the SC group requested feedback only in 7% of the practice trials [37]. In addition, Chiviacowsky and Wulf reported that feedback was requested on 44.7% of practice trials in the first practice block, but this decreased to 28% in the final practice block [40]. In contrast, in the present study, the frequency of feedback hardly decreased over practice. Wulf and Shea reported that 100% feedback, where feedback is given on every trial, is more effective for learning complex motor tasks than 50% feedback, where feedback is given every other trial [60]. Therefore, it is possible that the effect of practicing with high-frequency feedback was greater for low-skilled physical therapy students than the effect of SC of feedback, leading to similar learning effects in both groups.

Second, regarding the accuracy of visual estimation, the results of this study showed that similar to measurements using a universal goniometer, the accuracy of visual estimation improved with practice, and this skill was maintained even the day after practice. The improved visual estimation accuracy in both groups might have influenced the improvement in measurement techniques using the universal goniometer. Kantak and Winstein emphasized the importance of encoding processes such as error estimation during practice in motor learning [61]. Error estimation during practice is reportedly effective for learning if feedback is continuously provided [62]. In this study, because the SC group requested feedback frequently, both groups performed goniometric and visual estimation tasks with frequent feedback. By receiving frequent feedback on a task for which they had previously not received any feedback, participants processed information more accurately compared with their previous in-school practice without feedback. In addition, it is argued that the learners in both groups developed error detection ability (error estimation) and could modify motor programs more precisely. Consequently, the value of the feedback information provided was maximized by comparing the perceived error to the actual error, and the effect of SC was relatively small.

In addition to improving the accuracy of both the universal goniometer measurement and visual estimation, practice with feedback also shortened the measurement time for both methods. However, measurements using a universal goniometer require more time than visual estimation, likely because visual estimation does not use a universal goniometer; therefore, it does not take much time. Using visual estimation, the participants completed the measurement in a shorter time; however, as mentioned earlier, the measurement accuracy was lower than that using a universal goniometer. Hancock et al. reported that the visual estimation accuracy was lower than measurements using a universal goniometer among orthopedic surgeons, orthopedic trainees, and physical therapists [63]. Contrariwise, Blonna et al. examined the accuracy of angle measurements and visual estimation by four observers with different levels of experience; they reported that the accuracy of visual estimation made by experienced observers (experienced physicians and physician assistants) was superior to that of the accuracy of goniometer measurements made by less experienced observers (researchers and study coordinators) [25]. Although the physical therapy students who participated in this study improved the accuracy of their visual estimation through practice using feedback, the amount of practice and experience was still limited, and the difference in measurement accuracy with goniometric measurements remained unchanged. Watkins et al. suggested that ROM measurements of a patient’s knee should be performed repeatedly using a goniometer to minimize errors associated with the measurements [30]. For these reasons, measurement using a universal goniometer than visual estimation is recommended for students and other inexperienced healthcare professionals.

Regarding SE of ROM measurement, as reported in a previous study [48], SE before the pretest measurement did not reflect the accuracy of ROM measurement using a universal goniometer. However, for both groups, SE before the STRT measurement, immediately after practice, was related to measurement accuracy at that time. Normally, students do not know the actual ROM when learning techniques in lectures or through independent practice; therefore, it is difficult for them to understand their own skills correctly. However, in the present study, as physical therapy students correctly recognized their own measurement accuracy through practice using feedback, a relationship between SE before the STRT and the measurement accuracy of the test was determined. Regarding SE on the day after practice, only the SC group was associated with measurement accuracy immediately after practice; however, it did not reflect measurement accuracy on the day after practice. Self-regulation of the learning model was the reason SE on the day after practice was related to measurement accuracy immediately after practice only in the SC group. During the practice, the SC group reflected on the trials they had performed and repeatedly considered whether they needed to receive feedback. In other words, the three-stage cycle of forethought, performance control, and self-reflection in self-regulation learning was strengthened, and the SC group was able to process information more deeply. As a result, it is believed that the SC group formed SE that was appropriate to the situation of the STRT trial, even though the test trial was not given feedback. However, because the measurement accuracy changed considerably from immediately after practice to the day after practice, there was no relationship between SE and measurement accuracy on the day after practice. Baaji et al. reported that dental students are guaranteed a certain level of competency at the end of their undergraduate training; however, the level of SE varies from student to student and improves over a year as they gain experience after graduation [64]. In the SC group, by incorporating feedback into practice, students correctly recognized the measurement accuracy at that point; however, the amount of practice in this study possibly was insufficient for SE to fully reflect the measurement accuracy after learning.

Finally, the results of this study revealed that physical therapy students’ ROM measurement skills of universal goniometer measurement and visual estimation improved, and they learned through practice with feedback. However, even with improved measurement technique, visual estimation remains less accurate than measurements obtained using a universal goniometer. This study demonstrated the effectiveness of feedback as a method for acquiring ROM measurement techniques for physical therapy students.

### Limitations of the study

This study has several limitations. First, despite having calculated the sample size in advance, we were unable to recruit a sufficient number of participants. The post hoc power analysis using the sample size and effect size of this study indicated that the power was not sufficient (0.19). If the study results are based on an inadequate sample size, the results of statistical analyses may be overestimated. This means that it can be difficult to determine whether a study’s findings are true or whether they can be extrapolated to a larger population. Therefore, we propose conducting further research with a larger sample size to determine the motor learning effects of practice by using feedback on ROM measurement techniques.

Second, while this study only compared the SC group and the Yk group, studies examining the effects of SC feedback may compare them with a control group for which no feedback is given, or a 100% feedback group in which feedback is given on all trials [37, 42]. The results of this study showed that high-frequency feedback induced motor learning in both groups. However, since no comparison was made with 100% feedback, the most frequent feedback, interpretation of the results remains speculative. Incorporating control and 100% feedback groups could enhance the clarity of our results. In the future, in addition to increasing the number of participants, adding a control group and a 100% feedback group as new conditions will likely help to clarify more effective methods of feedback and gain insight into that feedback’s mechanisms.

Third, the instruments and methods used for evaluation present limitations. The tool employed to assess self-efficacy was adapted by our research team. Despite reference to previous research, the validity of this instrument, particularly the single-item SE scale, may still be questioned.

Finally, this study evaluated only knee flexion ROM measurements and focused on short-term practice effects; therefore, it remains unclear whether the findings can be generalized to measurements of other joints or whether long-term feedback impacts motor learning during ROM measurement practice. Therefore, in the future, the team plans to apply feedback-based training to measure joints other than the knee and verify its effects over a longer period.

## Conclusions

This study demonstrated that practice with external feedback improved the accuracy of students’ ROM measurements and visual estimation and shortened the measurement time. Furthermore, the results showed that the learning effect persisted after 24 h. However, the effectiveness of SC feedback could not be clarified. It was also revealed that SE after the end of the practice was temporarily related to measurement accuracy at that time. We believe that providing external feedback will lead to effective educational methods promoting the use of physical therapy techniques.

## Data Availability

No datasets were generated or analysed during the current study.

## Abbreviations

ROM: Range of Motion
SC: Self-controlled
Yk: Yoked
SE: Self-Efficacy
GMT: Goniometric Measurement Task
VET: Visual Estimation Task

## References

[1] Boyer KA, Johnson RT, Banks JJ, Jewell C, Hafer JF. Systematic review and meta-analysis of gait mechanics in young and older adults. Exp Gerontol. 2017;95:63–70. 28499954 10.1016/j.exger.2017.05.005

[2] Astephen JL, Deluzio KJ, Caldwell GE, Dunbar MJ. Biomechanical changes at the hip, knee, and ankle joints during gait are associated with knee osteoarthritis severity. J Orthop Res. 2008;26:332–41. 17960658 10.1002/jor.20496

[3] Monda M, Goldberg A, Smitham P, Thornton M, McCarthy I. Use of inertial measurement units to assess age-related changes in gait kinematics in an active population. J Aging Phys Act. 2015;23:18–23. 24306618 10.1123/japa.2012-0328

[4] Thaler-Kall K, Peters A, Thorand B, Grill E, Autenrieth CS, Horsch A, et al. Description of spatio-temporal gait parameters in elderly people and their association with history of falls: results of the population-based cross-sectional KORA-Age study. BMC Geriatr. 2015;15:32. 25880255 10.1186/s12877-015-0032-1 PMC4374293

[5] Benson LC, Cobb SC, Hyngstrom AS, Keenan KG, Luo J, O’Connor KM. Identifying trippers and non-trippers based on knee kinematics during obstacle-free walking. Hum Mov Sci. 2018;62:58–66. 30245267 10.1016/j.humov.2018.09.009

[6] Hyodo K, Masuda T, Aizawa J, Jinno T, Morita S. Hip, knee, and ankle kinematics during activities of daily living: a cross-sectional study. Braz J Phys Ther. 2017;21:159–66. 28473283 10.1016/j.bjpt.2017.03.012 PMC5537477

[7] García-Pinillos F, Ruiz-Ariza A, Moreno del Castillo R, Latorre-Román PÁ. Impact of limited hamstring flexibility on vertical jump, kicking speed, sprint, and agility in young football players. J Sports Sci. 2015;33:1293–7. 25761523 10.1080/02640414.2015.1022577

[8] Mills M, Frank B, Goto S, Blackburn T, Cates S, Clark M, et al. Effect of restricted hip flexor muscle length on hip extensor muscle activity and lower extremity biomechanics in college-aged female soccer players, Int J Sports Phys Ther. 2015;10:946–54. PMC4675195 26673683

[9] Nunome H, Ikegami Y, Kozakai R, Apriantono T, Sano S. Segmental dynamics of soccer instep kicking with the preferred and non-preferred leg. J Sports Sci. 2006;24:529–41. 16608767 10.1080/02640410500298024

[10] Bradley PS, Portas MD. The relationship between preseason range of motion and muscle strain injury in elite soccer players. J Strength Cond Res. 2007;21:1155–9. 18076233 10.1519/R-20416.1

[11] Henderson G, Barnes CA, Portas MD. Factors associated with increased propensity for hamstring injury in english premier league soccer players. J Sci Med Sport. 2010;13:397–402. 19800844 10.1016/j.jsams.2009.08.003

[12] Witvrouw E, Danneels L, Asselman P, D’Have T, Cambier D. Muscle flexibility as a risk factor for developing muscle injuries in male professional soccer players. A prospective study. Am J Sports Med. 2003;31:41–6. 12531755 10.1177/03635465030310011801

[13] Peters PG, Herbenick MA, Anloague PA, Markert RJ, Rubino LJ. Knee range of motion: reliability and agreement of 3 measurement methods. Am J Orthop (Belle Mead NJ). 2011;40:E249–52. 22268016

[14] Lavernia C, D’Apuzzo M, Rossi MD, Lee D. Accuracy of knee range of motion assessment after total knee arthroplasty. J Arthroplasty. 2008;23(Suppl 1):85–91. 18722308 10.1016/j.arth.2008.05.019

[15] Kato M, Echigo A, Ohta H, Ishiai S, Aoki M, Tsubota S et al. The accuracy of goniometric measurements of proximal interphalangeal joints in fresh cadavers: comparison between methods of measurement, types of goniometers, and fingers. J Hand Ther. 2007;20:12– 8; quiz 19. 10.1197/j.jht.2006.11.015 17254904

[16] Bronner S, Agraharasamakulam S, Ojofeitimi S. Reliability and validity of electrogoniometry measurement of lower extremity movement. J Med Eng Technol. 2010;34:232–42. 20180734 10.3109/03091900903580512

[17] Zampagni ML, Casino D, Zaffagnini S, Visani AA, Marcacci M. Estimating the elbow carrying angle with an electrogoniometer: acquisition of data and reliability of measurements. Orthopedics. 2008;31:370. 19292279 10.3928/01477447-20080401-39

[18] Goodwin J, Clark C, Deakes J, Burdon D, Lawrence C. Clinical methods of goniometry: a comparative study. Disabil Rehabil. 1992;14:10–5. 1586755 10.3109/09638289209166420

[19] Lenssen AF, van Dam EM, Crijns YHF, Verhey M, Geesink RJT, van den Brandt PA, et al. Reproducibility of goniometric measurement of the knee in the in-hospital phase following total knee arthroplasty. BMC Musculoskelet Disord. 2007;8:83. 17705860 10.1186/1471-2474-8-83 PMC2040146

[20] Rachkidi R, Ghanem I, Kalouche I, El Hage S, Dagher F, Kharrat K. Is visual Estimation of passive range of motion in the pediatric lower limb valid and reliable? BMC Musculoskelet Disord. 2009;10:126. 19822011 10.1186/1471-2474-10-126 PMC2765954

[21] Terwee CB, de Winter AF, Scholten RJ, Jans MP, Devillé W, van Schaardenburg D, et al. Interobserver reproducibility of the visual Estimation of range of motion of the shoulder. Arch Phys Med Rehabil. 2005;86:1356–61. 16003664 10.1016/j.apmr.2004.12.031

[22] van de Pol RJ, van Trijffel E, Lucas C. Inter-rater reliability for measurement of passive physiological range of motion of upper extremity joints is better if instruments are used: a systematic review. J Physiother. 2010;56:7–17. 20500132 10.1016/s1836-9553(10)70049-7

[23] Tojima M, Ogata N, Yozu A, Sumitani M, Haga N. Novel 3-dimensional motion analysis method for measuring the lumbar spine range of motion: repeatability and reliability compared with an electrogoniometer. Spine. 2013;38:E1327–33. 23797505 10.1097/BRS.0b013e3182a0dbc5

[24] Edwards JZ, Greene KA, Davis RS, Kovacik MW, Noe DA, Askew MJ. Measuring flexion in knee arthroplasty patients. J Arthroplasty. 2004;19:369–72. 15067653 10.1016/j.arth.2003.12.001

[25] Blonna D, Zarkadas PC, Fitzsimmons JS, O’Driscoll SW. Accuracy and inter-observer reliability of visual Estimation compared to clinical goniometry of the elbow. Knee Surg Sports Traumatol Arthrosc. 2012;20:1378–85. 22089371 10.1007/s00167-011-1720-9

[26] Menadue C, Raymond J, Kilbreath SL, Refshauge KM, Adams R. Reliability of two goniometric methods of measuring active inversion and Eversion range of motion at the ankle. BMC Musculoskelet Disord. 2006;7:60. 16872545 10.1186/1471-2474-7-60 PMC1550229

[27] Youdas JW, Bogard CL, Suman VJ. Reliability of goniometric measurements and visual estimates of ankle joint active range of motion obtained in a clinical setting. Arch Phys Med Rehabil. 1993;74:1113–8. 8215866 10.1016/0003-9993(93)90071-h

[28] Brosseau L, Balmer S, Tousignant M, O’Sullivan JP, Goudreault C, Goudreault M, et al. Intra- and intertester reliability and criterion validity of the parallelogram and universal goniometers for measuring maximum active knee flexion and extension of patients with knee restrictions. Arch Phys Med Rehabil. 2001;82:396–402. 11245764 10.1053/apmr.2001.19250

[29] Brosseau L, Tousignant M, Budd J, Chartier N, Duciaume L, Plamondon S, et al. Intratester and intertester reliability and criterion validity of the parallelogram and universal goniometers for active knee flexion in healthy subjects. Physiother Res Int. 1997;2:150–66. 9421820 10.1002/pri.97

[30] Watkins MA, Riddle DL, Lamb RL, Personius WJ. Reliability of goniometric measurements and visual estimates of knee range of motion obtained in a clinical setting. Phys Ther. 1991;71:90– 6; discussion 96– 7. 10.1093/ptj/71.2.90 1989012

[31] Gajdosik RL, Bohannon RW. Clinical measurement of range of motion. Review of goniometry emphasizing reliability and validity. Phys Ther. 1987;67:1867–72. 3685114 10.1093/ptj/67.12.1867

[32] Akizuki K, Yamaguchi K, Morita Y, Ohashi Y. The effect of proficiency level on measurement error of range of motion. J Phys Ther Sci. 2016;28:2644–51. 27799712 10.1589/jpts.28.2644 PMC5080194

[33] Rose V, Nduka CC, Pereira JA, Pickford MA, Belcher HJ. Visual Estimation of finger angles: do we need goniometers? J Hand Surg Br. 2002;27:382–4. 12162984 10.1054/jhsb.2002.0782

[34] Schmidt RA, Lee TD. Motor learning and performance: from principles to application. J Hum Kinet. 2019:249–74.

[35] Akizuki K, Mitamura K, Yamamoto R, Yamaguchi K, Ohashi Y. Extrinsic feedback from a feedback device promotes the learning of range of motion measurements. J Phys Ther Sci. 2020;32:114–9. 32158073 10.1589/jpts.32.114 PMC7032977

[36] Janelle CM, Barba DA, Frehlich SG, Tennant LK, Cauraugh JH. Maximizing performance feedback effectiveness through videotape replay and a self-controlled learning environment. Res Q Exerc Sport. 1997;68:269–79. 9421839 10.1080/02701367.1997.10608008

[37] Janelle CM, Kim J, Singer RN. Subject-controlled performance feedback and learning of a closed motor skill. Percept Mot Skills. 1995;81:627–34. 8570369 10.1177/003151259508100253

[38] Wulf G, Clauss A, Shea CH, Whitacre CA. Benefits of self-control in dyad practice. Res Q Exerc Sport. 2001;72:299–303. 11561396 10.1080/02701367.2001.10608964

[39] Chen D, Hendrick JL, Lidor R. Enhancing self-controlled learning environments: the use of self-regulated feedback information. J Hum Mov Stud. 2002;43:69–86.

[40] Chiviacowsky S, Wulf G. Self-controlled feedback: does it enhance learning because performers get feedback when they need it? Res Q Exerc Sport. 2002;73:408–15. 12495242 10.1080/02701367.2002.10609040

[41] Chiviacowsky S, Wulf G. Self-controlled feedback is effective if it is based on the learner’s performance. Res Q Exerc Sport. 2005;76:42–8. 15810769 10.1080/02701367.2005.10599260

[42] Sheaves EG, Snodgrass SJ, Rivett DA. Learning lumbar spine mobilization: the effects of frequency and self-control of feedback. J Orthop Sports Phys Ther. 2012;42:114–24. 10.2519/jospt.2012.3691. Epub 2011 Oct 25. PMID: 22030595. 22030595 10.2519/jospt.2012.3691

[43] Zimmerman BJ. Attainment of self-regulation: a social cognitive perspective. In: Boekarts M, Pintrich PR, Zeidner M, editors. Handbook of self-regulation. San Diego, CA: Academic; 2000. pp. 13–39.

[44] Kok M, Komen A, van Capelleveen L, van der Kamp J. The effects of self-controlled video feedback on motor learning and self-efficacy in a physical education setting: an exploratory study on the shot-put. Phys Educ Sport Pedagogy. 2020;25:49–66. 10.1080/17408989.2019.1688773.

[45] Ste-Marie DM, Carter MJ, Law B, Vertes K, Smith V. Self-controlled learning benefits: exploring contributions of self-efficacy and intrinsic motivation via path analysis. J Sports Sci. 2016;34:1650–6. 10.1080/02640414.2015.1130236. 26707002 10.1080/02640414.2015.1130236

[46] Bandura A. Self-efficacy: toward a unifying theory of behavioral change. Psychol Rev. 1977;84:191–215. 847061 10.1037//0033-295x.84.2.191

[47] Morton J, Anderson L, Frame F, Moyes J, Cameron H. Back to the future: teaching medical students clinical procedures. Back Future Med Teach. 2006;28:723–8. 17594585 10.1080/01421590601110025

[48] Yamamoto R, Yoshizato Y, Imai T, Akizuki K. Effect of the post-learning period on the accuracy and self-efficacy of measuring the joint range of motion. J Phys Ther Sci. 2023;35:708–13. 37791003 10.1589/jpts.35.708 PMC10542422

[49] Ste-Marie DM, Vertes KA, Law B, Rymal AM. Learner-controlled self-observation is advantageous for motor skill acquisition. Front Psychol. 2013;3:1–10. 10.3389/fpsyg.2012.00556. 10.3389/fpsyg.2012.00556 PMC3554505 23355826

[50] Islam R, Bennasar M, Nicholas K, Button K, Holland S, Mulholland P, et al. A nonproprietary movement analysis system (MoJoXlab) based on wearable inertial measurement units applicable to healthy participants and those with anterior cruciate ligament reconstruction across a range of complex tasks: validation study. JMIR MHealth UHealth. 2020;8:e17872. 32543446 10.2196/17872 PMC7327594

[51] Johnston KN, Young M, Kay D, Booth S, Spathis A, Williams MT. Attitude change and increased confidence with management of chronic breathlessness following a health professional training workshop: a survey evaluation. BMC Med Educ. 2020;20:90. 32228544 10.1186/s12909-020-02006-7 PMC7106669

[52] Tran K, Kovalskiy A, Desai A, Imran A, Ismail R, Hernandez C. The effect of volunteering at a student-run free healthcare clinic on medical students’ self-efficacy, comfortableness, attitude, and interest in working with the underserved population and interest in primary care. Cureus. 2017;9:e1051. 28367389 10.7759/cureus.1051 PMC5364087

[53] Gist ME, Mitchell TR. Self-efficacy: A theoretical analysis of its determinants and malleability. Acad Manag Rev. 1992;17:183.

[54] Medina-Ramírez RI, Álamo-Arce DD, Rodriguez-Castro F, Cecilio-Fernandes D, Sandars J, Costa MJ. Self-regulated learning microanalysis for the study of the performance of clinical examinations by physiotherapy students. BMC Med Educ. 2020;20:233. 32698789 10.1186/s12909-020-02149-7 PMC7374893

[55] Seitz AR, Nanez JE, Sr, Holloway S, Tsushima Y, Watanabe T. Two cases requiring external reinforcement in perceptual learning. J Vis. 2006;22:966–73. 10.1167/6.9.9 17083288

[56] Herzog MH, Fahle M. The role of feedback in learning a Vernier discrimination task. Vis Res. 1997;37:2133–41. 9327060 10.1016/s0042-6989(97)00043-6

[57] Wulf G, Iwatsuki T, Machin B, Kellogg J, Copeland C, Lewthwaite R. Lassoing skill through learner choice. J Mot Behav. 2018;50:285–92. 28854061 10.1080/00222895.2017.1341378

[58] Carter MJ, Ste-Marie DM. Not all choices are created equal: task-relevant choices enhance motor learning compared to task-irrelevant choices. Psychon Bull Rev. 2017;24:1879–88. 28224481 10.3758/s13423-017-1250-7

[59] St Germain L, Williams A, Balbaa N, Poskus A, Leshchyshen O, Lohse KR, et al. Increased perceptions of autonomy through choice fail to enhance motor skill retention. J Exp Psychol Hum Percept Perform. 2022;48:370–9. 35201814 10.1037/xhp0000992

[60] Wulf G, Shea CH, Matschiner S. Frequent feedback enhances complex motor skill learning. J Mot Behav. 1998;30:180–92. 20037033 10.1080/00222899809601335

[61] Kantak SS, Winstein CJ. Learning-performance distinction and memory processes for motor skills: a focused review and perspective. Behav Brain Res. 2012;228:219–31. 22142953 10.1016/j.bbr.2011.11.028

[62] Guadagnoli MA, Kohl RM. Knowledge of results for motor learning: relationship between error Estimation and knowledge of results frequency. J Mot Behav. 2001;33:217–24. 11404216 10.1080/00222890109603152

[63] Hancock GE, Hepworth T, Wembridge K. Accuracy and reliability of knee goniometry methods. J Exp Orthop. 2018;5:46. 30341552 10.1186/s40634-018-0161-5 PMC6195503

[64] Baaij A, Özok AR, Vӕth M, Musaeus P, Kirkevang LL. Self-efficacy of undergraduate dental students in endodontics within Aarhus and Amsterdam. Int Endod J. 2020;53:276–84. 31519031 10.1111/iej.13218 PMC7006807

